# Supplementary material for: Genetic consequences of improved river connectivity in brown trout (Salmo trutta L.)
Source: Evol Appl. 2024 Apr 11;17(4):e13660. doi: 10.1111/eva.13660 (PMC11009428; doi:10.1111/eva.13660)
Supplement: Supplementary file 1 — Data S1: Supporting Information. [file EVA-17-e13660-s001.pdf]

Supplementary material:

Genetic consequences of improved river connectivity in brown trout (*Salmo trutta* L.)

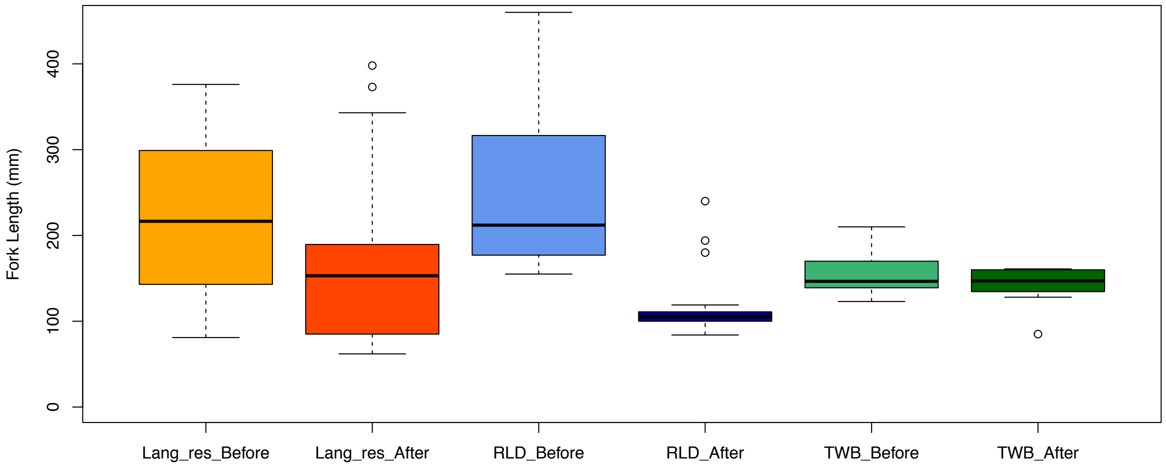

Figure S1: Size distribution of brown trout captured at Langsett reservoir system before and after the fish pass construction.

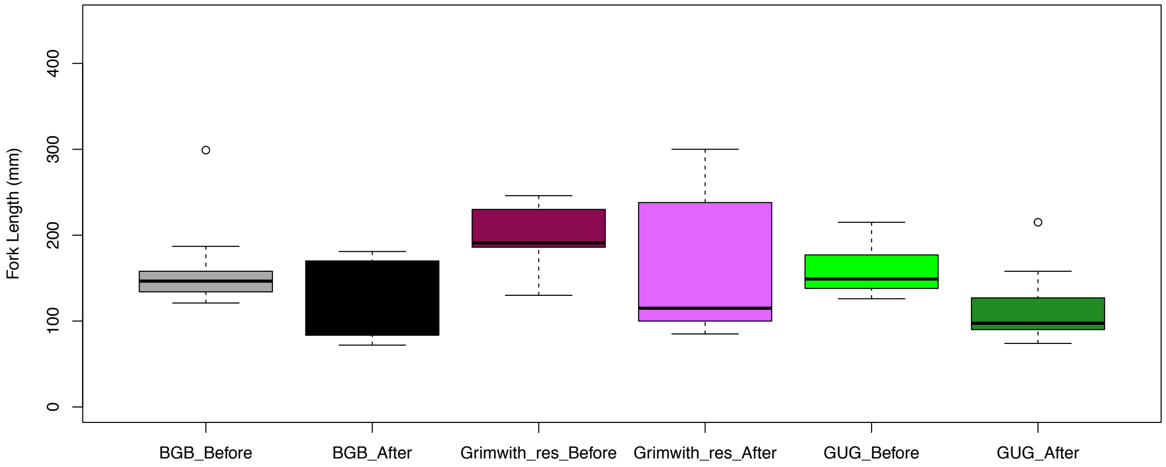

Figure S2. Size distribution of brown trout captured at Grimwith reservoir system before and after the fish pass construction.

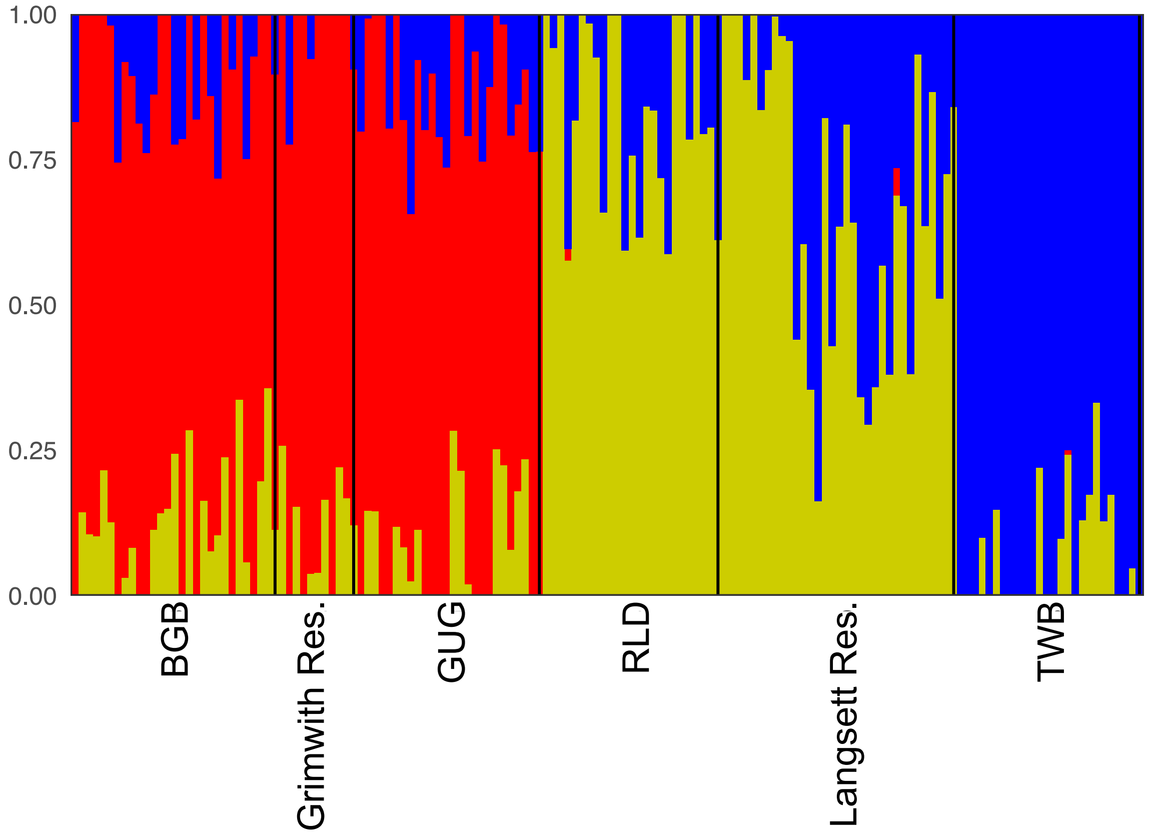

Figure S3: ADMIXTURE analysis plot (K=3) of brown trout populations from Langsett and Grimwith reservoir systems prior to fish pass opening on the RLD. The analysis is based on 81 SNPs. Colours indicate different genetic clusters. Each fish individual is represented by a vertical bar.

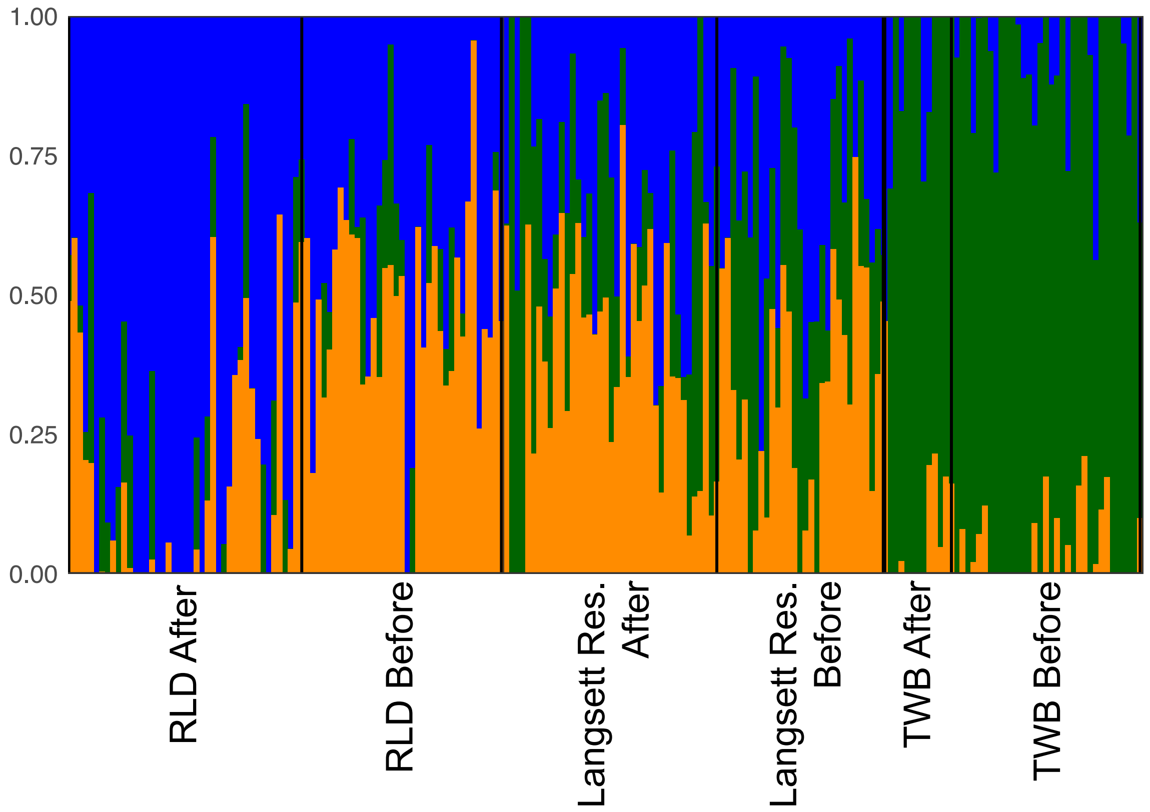

Figure S4: ADMIXTURE analysis plot (K=3) of brown trout populations from Langsett reservoir system before and after the fish pass opening on the RLD. The analysis is based on 81 SNPs. Colours indicate different genetic clusters. Each fish individual is represented by a vertical bar. An analogous analysis conducted on Grimwith reservoir system revealed absence of structuring within the system (K=1 most likely number of genetic units), therefore no figure was generated.

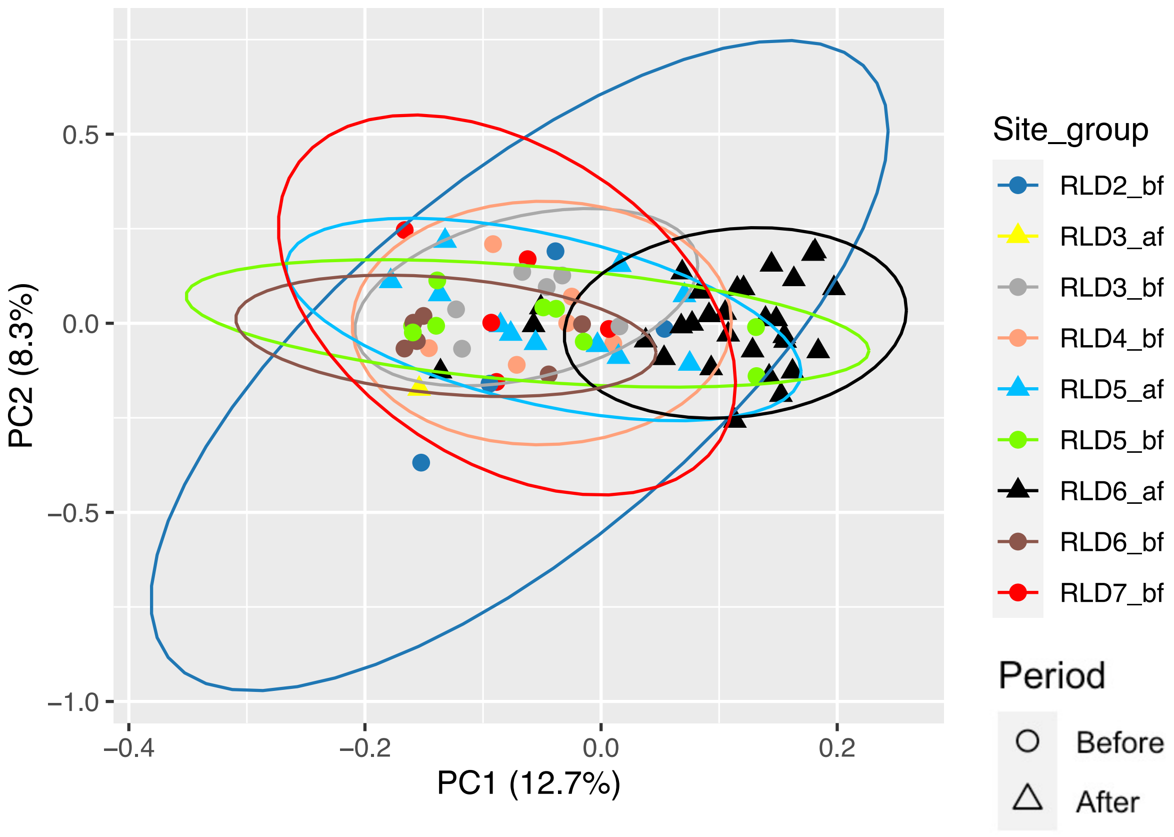

Figure S5. Genetic structuring of brown trout populations from the River Little Don (RLD) before and after fish construction. The principal components analysis (PCA) plot is based on 81 SNPs. Dots represent individual fish and are coloured based on sampling site and period (bf = before, af = after). Variance (%) explained by the first and second axes is shown.

Table S1: Samples caught at each sampling location at Langsett reservoir system with geographic coordinates.

			Location	
**Lat.**	**Long.**	**Site ID**	**RLD Before**	**RLD After**
53.49687	-1.71360	RLD2	4	0
53.49818	-1.71359	RLD3	6	1
53.49947	-1.71274	RLD4	6	0
53.50085	-1.71101	RLD5	9	11
53.50142	-1.70834	RLD6	6	30
53.50169	-1.70611	RLD7	5	0
		**Total**	36	42
			**Lang Res Before**	**Lang Res After**
Various sites		Main reservoir	13	6
53.50180	-1.70163	Downstream RLD weir	16	32
53.49125	-1.68776	Downstream TWB weir	1	1
		**Total**	30	39
			**TWB Before**	**TWB After**
53.48475	-1.68778	TWB1	1	1
53.48586	-1.68816	TWB2	2	0
53.48730	-1.68681	TWB3	6	0
53.48825	-1.68697	TWB4	8	0
53.48925	-1.68687	TWB5	8	9
53.49007	-1.68736	TWB6	9	2
		**Total**	34	12

Table S2: Samples caught at each sampling location at Grimwith reservoir system with geographic coordinates.

			Location	
**Lat.**	**Long.**	**Site ID**	**BGB Before**	**BGB After**
54.08865	-1.92784	BGB1	14	4
54.08816	-1.92629	BGB2	7	4
54.08724	-1.92507	BGB3	6	4
		NA	3	0
		**Total**	30	12
			**Grim Res Before**	**Grim Res After**
Various sites		Main reservoir	12	16
		**Total**	12	16
			**GUG Before**	**GUG After**
54.09082	-1.91970	GUG1	7	4
54.08961	-1.91947	GUG2	12	4
54.08822	-1.91951	GUG3	10	4
		**Total**	29	12

Table S3. Proportions of successful reassignment (based on DAPC) of individual brown trout to their original groups.

**Location**	**Proportion of successful reassignment**
BGB Before	50.0 %
BGB After	0 %
Grimwith Reservoir Before	0 %
Grimwith Reservoir After	18.8 %
GUG Before	41.4 %
GUG After	0 %
RLD Before	52.8 %
RLD After	73.8 %
Langsett Reservoir Before	0 %
Langsett Reservoir After	43.6%
TWB Before	97.1 %
TWB After	0 %
